# P08 Antibiotic residue higher in pre-filled compared with self-filled B Braun Easypump II devices

**DOI:** 10.1093/jacamr/dlaf239.012

**Published:** 2026-01-05

**Authors:** Nicola Walker, Daniel Hearsey, Jade Lee-Milner, Claire Painter, Stuart E Bond

**Affiliations:** Mid Yorkshire Teaching NHS Trust, Wakefield, UK; Royal Cornwall Hospital NHS Trust, Cornwall, UK; Mid Yorkshire Teaching NHS Trust, Wakefield, UK; Acute Care at Home, Truro Health Park, Infirmary Hill, Truro, UK; Mid Yorkshire Teaching NHS Trust, Wakefield, UK

## Abstract

**Background:**

The three options for elastomeric antibiotic pumps (EMP) used in patient’s homes are: pre-filled by a commercial manufacturer, pre-filled by an NHS pharmacy aseptic unit, and filled by nurses at the point of care. Mid Yorkshire Teaching NHS Trust (MYTT) uses commercially pre-filled Easypump^®^ II EMPs for piperacillin/tazobactam and flucloxacillin. Royal Cornwall Hospitals NHS Trust (RCHT) and Cornwall Partnership NHS Foundation Trust (CPFT) nurse-fill Easypump^®^ II EMPs in the patient’s home. Commercially filled devices are the most costly of the filling options. Representatives from the three NHS Trusts performed a joint audit of antibiotic EMP residue.

**Objectives:**

To compare EMP residue in MYTT pre-filled Easypump^®^ II devices with CPFT nurse-filled Easypump^®^ II devices.

**Methods:**

The quality improvement project did not require ethics approval. For two weeks in February and March 2025, MYTT community nurses collected all administered piperacillin/tazobactam and flucloxacillin EMPs. The specialist pharmacy technician drained the EMPs and measured the residue. The CPFT OPAT nurses weighed each administered pump and used the weight of an empty pump to calculate the antibiotic residue. An Excel spreadsheet was used to document the drug, dose, type of vascular access, pre-filled/self-filled and antibiotic residue. Using Microsoft Excel^®^, a two-proportion z-test was used to determine statistical significance and a Mann–Whitney *U*-test was used to compare the EMP residual volumes.

**Results:**

MYTT collected 84 EMPs which included 9 piperacillin/tazobactam 13.5 g, 45 piperacillin/tazobactam 18 g, 28 flucloxacillin 8 g and 2 flucloxacillin 12 g. CPFT collected 55 EMPs which included 11 piperacillin/tazobactam 13.5 g, 24 piperacillin/tazobactam 18 g and 20 flucloxacillin 8 g. Twelve results were excluded (10 for MYTT, 2 for CPFT) due to known pump or vascular access issues. More pre-filled than nurse-filled EMPs contained residue (MYTT median residue 29 mL versus CPFT 17 mL, *P*=0.04). More piperacillin/tazobactam than flucloxacillin EMPs contained residue (median 30 mL versus 8 mL, *P*=0.008). No difference in residue was found between strengths of piperacillin/tazobactam, between strengths of flucloxacillin, or between vascular devices.

**Conclusions:**

Pre-filled Easypump^®^ II EMPs were more likely to have residue than nurse-filled Easypump^®^ II EMPs. A possible reason for this is crystallization in the pump due to longer expiry. Antibiotic residue was also more likely with piperacillin/tazobactam than flucloxacillin. The method of measuring residue should be changed to be identical for both sites, such as weighing the EMP. Our findings will be used to inform future service improvement, and the design of a larger project is recommended across multiple Trusts to validate results.

